# Transcriptomic Analysis of Glycosylation and Neuroregulatory Pathways in Rodent Models in Response to Psychedelic Molecules

**DOI:** 10.3390/ijms24021200

**Published:** 2023-01-07

**Authors:** Anup M. Oommen, Katherine J. Roberts, Lokesh Joshi, Stephen Cunningham

**Affiliations:** 1 Advanced Glycoscience Research Cluster (AGRC), University of Galway, H91 W2TY Galway, Ireland; 2 CÚRAM, SFI Research Centre for Medical Devices, Biomedical Sciences, University of Galway, H91 W2TY Galway, Ireland; 3 Department of Health and Behaviour Studies, Teachers College, Columbia University, New York, NY 10027, USA

**Keywords:** psychedelic, glycosylation, glycoconjugates, enrichment analysis, neuro-regulatory, molecular pathways

## Abstract

The potential for psychedelic molecules in impacting cognitive flexibility has long been supported and acknowledged across scientific reports. In the current study, an approach leveraging knowledge-based gene-set information analysis has been adopted to explore the potential impact of psychedelic molecules on both glycosylation, (a post-translational modifications (PTM)) and on neuro-regulatory pathways. Though limitations and restrictions rise from the scarcity of publicly available ‘omics’ data, targeted analysis enabled us to identify a number of key glycogenes (*Hexb*, *Hs6st2*, *Col9a2*, *B3gat2*, *Mgat5*, *Bgn*) involved the structural organization of extracellular matrix and neuroprotective factors (*Kl*, *Pomc*, *Oxt*, *Gal*, *Avp*, *Cartpt*) which play vital roles in neuron protection, development as well as synaptic stability. In response to psychedelic molecules, we found that these genes and associated pathways are transcriptional altered in rodent models. The approach used indicates the potential to exploit existing datasets for hypothesis generation and testing for the molecular processes which play a role in the physiological response to psychedelic molecule effects. These reported findings, which focused on alterations in glycogenes and neuro-regulatory factors may provide a novel range of biomarkers to track the beneficial, as well as potential toxicological effects of psychedelic molecules.

## 1. Introduction

Psychedelics, often termed as psychedelic drugs, hallucinogens, or hallucinogenic drugs represent a class of chemical substances that exert psycho-biological actions including alterations in consciousness, time perceptions, and visual perceptions as well as numerous cognitive processes [1]. Human usage of these chemical substances dates back to centuries or perhaps millennia and was identified as being used largely as a part of religious or recreational ceremonies across various parts of the world [2]. Despite the public perception of these substances beginning in 1960s as dangerous and the categorization of psychedelics as Schedule I substances by the 1967 UN Convention on Drugs, biologically they are the safest known class of substances that were developed as Central Nervous System (CNS) drugs for psychotherapy [3,4,5,6].

The major site of molecular action of psychedelic substances, including both classic as well as psychedelic-like molecules, involve the human brain, primarily targeting the serotonin and dopamine neurotransmitter signaling, which in turn affects the configuration and activity of functional brain connectivity networks [7,8,9] (Table 1). Modulation of these pathways, expressed on the excitatory and inhibitory neuronal circuits can induce hallucinogenic like effects.

Understanding the molecular mechanisms of action of a psychedelic molecule in conjunction with its impact on the brain’s cognitive functions could help explain the underlying ‘reasons’ for complex and variable results observed among subjects [20]. In certain neurotic disorders where low serotonin activity is a causal mechanism, such as depression and anxiety, treatment with psychedelic substances (LSD, DMT and psilocybin) has been shown to provide anti-depressant activity [21,22]. Further, a recent large scale clinical trial indicated strong positive results for the use of MDMA as a treatment option for post-traumatic stress disorder, PTSD [23]. Collectively, these studies highlight the detailed understanding of the complex interacting pathways underlying diverse neuro-regulatory circuits is essential for delineating the beneficial effects of psychedelic substances devoted to the treatment of diverse mental disorders.

However, this field is currently limited owing to the Schedule 1 classification of psychedelics [24] and the minimal data which exists for human usage. A need exists for multi-center clinical trials to be undertaken, aligned with the best clinical practices. Requirements of inclusion, monitoring and outcome measurements need to be established and a clear understanding of psychedelic agent dosages set in principal. Emerging neuropsychopharmacological studies of psychedelic molecules are helping to unravel the molecular mechanisms underlying the physiological response to these compounds [22,25,26,27,28,29,30,31,32]. These studies have generated datasets, which are now widely available for in depth analysis and hypothesis interrogation exploring the molecular functioning of the tested psychedelics and the biological alterations and processes they influence.

Within biological systems, post-translational modifications (PTMs) are processes which alter the properties of a protein and lipid molecules by proteolytic cleavage and addition of modifying group, such as acetyl, phosphoryl, glycosyl and methyl, to one or more amino acid position. PTMs play a key roles across numerous biological processes by significantly affecting the structure, function and dynamics of either molecule. Such modifications affect a wide range of biological behaviors and characteristics, including enzyme function and assembly, protein lifespan, protein–protein interactions, cell–cell and cell–matrix interactions, molecular trafficking, receptor activation, protein solubility, protein folding and protein localization.

Greater than 70% of brain proteins and lipids being PTMs by saccharide or glycan structures (collectively termed as glycoconjugates covering glycolipids, proteoglycans and glycoproteins) [33]. These glycoconjugates are known to play a vital role in brain development, myelination as well as the functioning of the synapses and neurotransmitters [34,35,36]; glycosylation remains unexplored as a molecular process directly involved in psychedelic agents. Research studies in both rodent and human species have indicated that saccharides (glycans) and glycoconjugates have a direct function and provide beneficial effects on cognitive performance and mental health, including mood and well-being [reviewed in [37,38,39,40]. Emerging research evidences establish the fact that environmental determinants such as stress, trauma and anxiety have profound long lasting effect on changing the mammalian glycan profile even in the brain tissues [41,42,43]. However, across literature, no studies to date have explored the impact of psychedelic substances on brain or systemic glycomic profile and its correlation with the associated psychedelic effects.

Within this study we leverage current transcriptomic data from open source gene expression databases to understand the transcriptional level changes of the genes involved in glycosylation pathways which assist in the synthesis of glycoconjugate structures as well as the glycan binding proteins (together, referred to as glycogenes from here onwards). Though it is challenging to directly interpret the glycan structure variability from transcriptomics data, owing to its non-template driven synthesis mechanism, recent study by Williams SE et al., has shown that mammalian brain glycan signature correlates with the RNA expression of the glycogenes [44]. Thus, the functional enrichment analysis of the differentially expressed glycogenes together with the neuro-regulatory markers may provide novel insights into the complex biological mechanisms in the brain regions that may correlate with the biopsychosocial aspects observed in response to psychedelic substances.

## 2. Results

### 2.1. Differentially Expressed Glycogene Transcripts

Applying stringent filtering criteria (adjusted *p*-value (FDR, Benjamini-Hochberg ≤ 0.05) and tested *p*-value ≤ 0.05) did not return DEGs for the enrichment analysis. Applying a relaxed filtering criteria, based on tested *p*-value, and the resulting DEGs were filtered by using a log fold change cut-off value of 0.5 for both upregulated and downregulated DEGs in order to filter out lowly expressed genes which were maximally distributed within this log fold range (see Supplementary Figure S1). Altogether, nine data sets (eight from the blood and brain tissues and one data point from the heart tissue) were identified as having significant DEGs for subsequent analysis. One study (GSE68175), which aimed at exploring the acute cardiotoxicity effects of MDMA in heart tissue was omitted and not considered for subsequent transcriptomic analysis. Datasets from the human and the *Sus scofa* species (GSE74737 and GSE172074, respectively) did not yield significant coverage of DEGs belonging to the glycosylation and neuro-regulatory gene subsets and were filtered out from further analysis. Similarly, processed data from the LSD treated rodent brain samples did not yield significant DEGs belonging to these subsets. The final list of datasets post filtering criteria for the subsequent detailed transcriptomic analysis for the glycogene as well as neuro-regulatory pathway genes were from the rodent species treated with ketamine and phencyclidine compounds.

**Figure 1:**
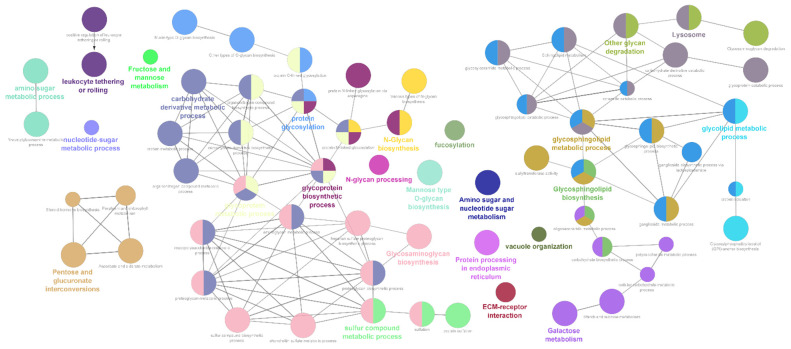
Gene ontology and pathway annotation network for the glycogene DEGs generated using the ClueGo v2.5.8 + CluePedia v.1.5.8 plugin via the Cytoscape software (v.3.9). The graph represents the enrichment analysis of the glycogene DEGs identified from the ketamine and phencyclidine treated rodent striatal and pre-limbic cortex tissues. The GO-Biological Process-EBI-Unirpot-GOA-ACAP-ARAP-13.05.2021, REACTOME PATHWAYS-13.05.2021 and KEGG-KEGG-13.05.2021 pathways were selected for the ontologies/Pathways and the evidence column was manually selected including the “All_Experimental”; RCA and TAS parameters. Pathways with *p* value ≤ 0.05 was selected for the filtering criteria and the CluePedia Option of five genes per term was selected for the visualization threshold. Network selectivity parameter was kept at Medium and the network connectivity Kappa score was set at 0.4.

In rodent species, out of the six datasets, maximum representation of glycogene DEGs were observed in the striatal tissue (see Supplementary Table S2). Broadly, the glycogene DEGs represented the pathways belonging to *N*-glycan and *O*-glycan biosynthesis, glycosphingolipid and glycosaminoglycan metabolic pathways along with the metabolic processes involved in the synthesis of substrates for glycan biosynthesis (Supplementary Table S3). A time dependent increase in representation of glycogene DEGs towards four and eight h in both ketamine and phencyclidine treated striatal tissues where observed (see Supplementary Table S2). Interestingly, these DEGs are largely represented by the glycosaminoglycan pathway including both proteoglycan and sulfated glycosaminoglycan biosynthesis processes (Figure 1). Furthermore, enrichment analysis of the data from the pre-limbic cortex treated with ketamine highlighted the differential regulation of mucin type *O*-glycan biosynthesis pathway in addition to the glycosaminoglycan metabolic processes (see Supplementary Table S2).

A significant observation among the glycogene DEGs is the differential expression of Klotho gene in the striatal tissues under both ketamine and phencyclidine treatment (Table 2). Both human correlational studies and mouse models of disease indicated Klotho as a critical neuroprotective factor against multiple neurological and psychological disorders [45]. Glycosyltransferases involved in the synthesis of sulfated carbohydrate epitope L2/HNK-1 expressed on several neural adhesion proteins and vital for cell–cell adhesion [46] were observed to be negatively regulated by the psychedelic molecules. A number of transcripts encoding sialyl- and glycosyltransferases involved in the glycosphingolipid biosynthetic pathways as well as sialylated *O*-linked glycans in brain that are vital for re-myelination, cell-adhesion and neuroglia differentiation [44] were found to be specifically enriched in the phencyclidine treated striatal tissue (Supplementary Table S6). Similarly, transcripts encoding glycosyltransferases as well as fucosyltransferases that are involved in the synthesis of *O*-mannosyl glycans and Lewis X antigens, respectively were found to be specifically enriched in the ketamine treated striatal tissue which play crucial role in cell adhesion and synaptic plasticity [47,48].

### 2.2. Differentially Expressed Neuro-Regulatory Transcripts

**Figure 2:**
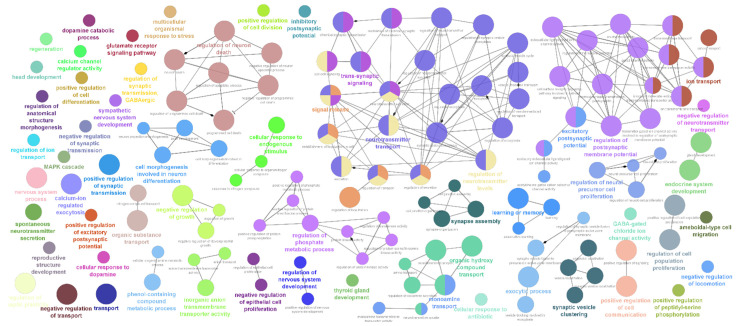
Gene ontology and pathway annotation network for the neuro-regulatory DEGs generated using the ClueGo v2.5.8 + CluePedia v.1.5.8 plugin via the Cytoscape software (v.3.9). The graph represents the enrichment analysis of the neuro-regulatory DEGs identified from the ketamine and phencyclidine treated rodent striatal tissue and the pre-limbic cortex. The GO-Biological Process-EBI-Unirpot-GOA-ACAP-ARAP-13.05.2021 was selected for the ontologies/Pathways and the evidence column was manually selected including the “All_Experimental”; RCA and TAS parameters. Pathways with *p* value ≤ 0.05 was selected for the filtering criteria and the CluePedia Option of five genes per term was selected for the visualization threshold. Network selectivity parameter was kept at Medium and the network connectivity Kappa score was set at 0.4.

Analysis of neuro-regulatory genes found a similar pattern as observed for the glycogene transcripts, a time dependent increase in the number of neuro-regulatory DEGs in the striatal tissue were observed for both ketamine and phencyclidine treatment (Supplementary Table S4). Interestingly, only a limited number of neuroendocrine secretory proteins, nerve growth factors and neurotrophic factors were differentially regulated in both ketamine and phencyclidine treatment (Supplementary Table S7). Gene annotation and pathway enrichment analysis revealed that these DEGs are involved in diverse biological processes which involve energy homeostasis, regulation of multiple hormone secretion, inflammatory response, neuro-synapse developmental process, organ development as well as multiple cognitive functions (Figure 2; Supplementary Table S5). Transcripts which encode proteins with neuropeptidal hormonal activity such as *Cartpt*, *Oxt*, *Avp* and *Gal* were found to be significantly upregulated in both ketamine and phencyclidine treatment (Table 2). This might implicate a complex biological pathway cascade emanating from the proteolytic processing of these precursor neuropeptides from these hormones which regulate diverse physiological processes such as addiction, stress response, anxiety, locomotor activity and energy homeostasis [49,50,51,52,53]. Acute treatment of both ketamine and phencyclidine was observed to decrease the expression of *Pomc* transcript whilst increased expression over long treatment time (Table 2). Interestingly, similar expression pattern was reported in a rodent study in response to MDMA treatment which was suggested to correlate with the rewarding effects of MDMA [49].

Furthermore, ketamine treatment is found to affect the differential expression of genes associated with the core biological processes such as regulation of postsynaptic membrane potential, regulation of neurotransmitter levels, dopamine metabolic process, regulation of neuron apoptotic process, neurotrophin signaling pathway as well as negative regulation of neuroblast proliferation. A time dependent increase in number of neuro-regulatory DEGs in the striatal tissue was a consistent pattern observed for both ketamine and phencyclidine treatment as noticed for the glycogene DEGs (Supplementary Table S7). Additionally, in the pre-limbic cortex, enrichment of vesicular trafficking and exocytic pathways as well as GABAergic synapse pathways were observed (Supplementary Table S7) which are vital neurotransmitter circuits modulating goal directed behavior, emotions as well as cognitive functions [54].

## 3. Discussion

A growing body of emerging reports and publications have indicated the great promise for psychedelic classes of drugs in treating mental-health conditions ranging from depression, trauma, addiction, social anxiety as well as eating disorders [6,45,46]. The profound measurable effects of psychedelic substances in the brain by modulating the prefrontal cortex-limbic system and the medial fronto-parietal network system [53] have been ascribed to underlie these observed therapeutic benefits. However, the biological mechanisms underlying this impact remains to be elucidated.

Apart from the therapeutic benefits, psychedelic molecules have also been reported to cause neurotoxic damage and long-lasting effects in the brain. For example, acute and repeated exposure to MDMA have been reported to induce long lasting changes in the brain reward-circuitry in rodents by altering the expression of opioid genes through transcriptional and post-translational modification mechanisms (PTMs) [55]. Among the PTMs, glycosylation modifications and the resulting complex glycoconjugate structures are vital regulators of neural transmission and excitability of neural circuits [56,57]. For example, the glycoconjugate structures such as proteoglycans as well as sulfated glycosaminoglycans are core biological structures within the neuronal extracellular matrix (ECM) [58,59] which play vital roles in neuron protection, development, as well as synaptic stability [60,61,62,63]. Diverse signaling molecules are generated from the neuronal ECM in response to multiple environmental stimuli and are reported to be critical for regulating synaptic plasticity, learning and memory.

Maximum representation of the glycosaminoglycan and proteoglycan biological processes in the striatal and pre-limbic cortex indicate the possibility of dynamic changes in the ECM molecules in response to the psychedelic substances induced hallucinogenic effects in these regions. Striatal tissue and the pre-limbic cortex (the rodent analogue for human dorsal anterior cingulate cortex) are regarded as the core regions underlying dopamine dependent neural circuits for hallucination like perception [64] and cholinergic dependent neural circuits for emotional as well as cognitive functions [65] in both rodent as well as human species. Differential regulation of glycosaminoglycan metabolism, including diverse proteoglycans, in response to the treatment of ketamine and phencyclidine may indicate a common underlying molecular mechanism mediating the mood-altering or hallucinogenic effects of these substances. The differentially expressed glycosyltransferases from both ketamine and phencyclidine treated samples were involved in pathways associated with the synthesis of sulfated carbohydrate epitopes, sialylated *O*-linked glycans, *O*-mannosyl glycans, Lewis X antigens and glycosphingolipid biosynthesis which play crucial role for re-myelination, cell-adhesion, neuroglia differentiation and synaptic plasticity [45,46,47,48]. One of the notable observation is the differential expression of *Kl* gene which encodes α-Klotho protein, a single pass type 1 integral membrane protein [66]. Owing to its role in diverse biological mechanisms such as activation of N-methyl-d-aspartate (NMDA) receptor signalling; stimulating the antioxidant defense system; reducing inflammation; promoting autophagy and enhancing clearance of amyloid-β [67], the glycoprotein Klotho is regarded as a critical neuroprotective factor against multiple neurological and psychological disorders [45].

Enrichment of biological processes causally linked to synaptic plasticity observed in the current study is consistent with previous reports which signifies that psychedelics promote the structural and functional plasticity of synapses [68]. Studies exploring the molecular mechanism of psychedelic substances induced mind altering effects, in both rodent and in vitro cell models, have also indicated the potential of neurotoxic effects for these molecules by inducing cytotoxicity, mitochondrial dysfunction and oxidative stress [69]. In this regard dose dependent neurotoxic effects of psychedelic molecules such as ketamine and phencyclidine have been previously reported in rodent models [70,71], and hence it is also important to consider whether biological processes associated with synaptic plasticity may be alter, enhanced or suppressed as a result of neuronal damage. Interestingly, the time dependent expression pattern of gene transcripts encoding for the neurotrophic factors that have neuroprotective functions, such as *Cartpt*, *Gal*, *Kl* and *Oxt* support this possibility [72,73,74,75]. Previous studies have also shown that the proteolytic processing of the precursor neuropeptides from CARTPT, Galanin, Oxytocin and Vasopressin have also been implicated in regulating diverse physiological processes such as addiction, stress response, anxiety, locomotor activity and energy homeostasis [49,50,51,52,53].

Interestingly, treatment with ketamine and phencyclidine had been shown to impact these physiological responses in humans pointing towards a potential of developing neuropeptide markers transcribed from *Cartpt, Oxt, AVP, Kl* and *Gal* as putative biomarkers to study the impact of psychedelic molecules on neurosynaptic plasticity as well as associated physiological responses observed in response to psychedelic treatment. In this regard, it is noteworthy to highlight the varying expression pattern observed for the *Pomc* gene transcript. Among the various peptides encoded by the *Pomc* gene transcript, β-endorphin has been shown to play a vital role in inducing euphoria as well as the rewarding and reinforcement properties of drug of abuse [76]. Further studies are needed to validate whether time dependent increase in expression of *Pomc* transcript correlate with increased levels of plasma β-endorphin levels in response to psychedelic treatment.

Owing to the current limited availability of complete ‘omics’ data resources, findings from the current study is restricted to data obtained from psychedelic substances tested in rodent species. Datasets identified from human failed to yield significant coverage of the knowledge based gene-sets explored. However, research studies suggest behavioral abnormalities observed in rodent species in response to psychedelic class of substances, bear the translational potential of predicting or inferring such effects in human. Therefore, the findings from this analysis may provide a framework to further explore the hypothesis of glycosylation and neuro-regulatory pathways as one of the biological mechanisms and source for molecular markers to explore the biopsychosocial aspects observed in response to psychedelic molecules.

It is important to note that the glycogenes observed to be differentially regulated in the ketamine and phencyclidine treatment belong to the glycosphingolipid pathways, ECM proteoglycans as well as sialylated and fucosylated *O*-glycan structures that are critical for modulating neuronal adhesion, myelination as well as synaptic plasticity [56]. This study also highlights the time dependent differential expression of diverse neuropeptidal hormones and receptors for neurotrophic factors (e.g., *Kl, Pomc, Oxt, Gal, Avp, Cartpt, Chrna10, and Sstr5*) that are implicated in the regulation of wide range of biological processes ranging from energy homeostasis to multiple cognitive functions. Interestingly, many of these proteins are also glycosylated (~48%), highlighting the critical need to evaluate whether psychedelic treatment could affect the stability, protein cleavage and processing of prepropeptides of these neuro-hormones by altering their glycosylation patterns [77].

Further research is required to validate such a hypothesis and assess the dynamic changes in ECM glycoconjugate structures as well as the glycosylated isoforms of neuropeptides to determine if they could act as putative biomarkers to investigate and explore the molecular mechanism of psychedelic induced hallucinogenic or neurotoxic effects in the brain. Moreover, detailed understanding of these biological pathways will support the differentiation of psychedelic substances, for the treatment and clinical use in post-traumatic stress disorders, major depression, anxiety disorders, addictions to compulsive substances, personality disorders and managing end-of-life symptoms.

## 4. Materials and Methods

### 4.1. Transcriptomic Data Selection, Processing and Differentially Expressed Genes (DEGs)

For transcriptomic analysis, public database Gene Expression Omnibus (GEO) (https://www.ncbi.nlm.nih.gov/geo/, accessed on 20 May 2021) was queried using the names of selected class of psychedelic molecules as the key words, (see Supplementary Table S1). Datasets generated from both human and non-human species were retrieved, and only datasets with ≥3 replicates for both test and control samples were included within this analysis. With the exception for one treatment study using methamphetamine (GSE130254), all other studies used intraperitoneal administration of the psychedelic molecules with varying doses ranging from 2 mg/kg to 40 mg/kg. Data processing and gene annotation were conducted using online software tools GenePattern (v3.9.11; http://software.broadinstitute.org/cancer/software/genepattern/ (accessed on 22 December 2022); Broad Institute, Massachusetts, and University of California, San Diego, CA, USA) [78] and the Galaxy web platform using the public server at usegalaxy.org (https://usegalaxy.org/ (accessed on 22 December 2022) (Galaxy Community Hub)) [79], following the analysis methodology and stringency levels as detailed in the software user guides. Significance of differential expressed genes were calculated using default *p*-value adjustment methods in analysis (*p* ≤ 0.05). Where available, datasets normalized with log2 transformed gene expression matrix files were used directly from the GEO databases. Summarizing fold distribution of differentially expressed genes was performed using the Analyze Data tool within Microsoft Excel. From these data points a matrix file was created in MS Excel by extracting the fold change and *p*-values for the targeted gene set analysis focusing only on the genes involved in glycosylation and the neuro-regulatory pathways. An additional processed gene expression data was kindly provided by Dr. Charles D Nichols, (Louisiana State University, School of Medicine, New Orleans, LA, USA) upon request, which studied the gene expression pattern of rodent brain in response to (+)-LSD tartrate treatment (1.0 mg/kg i.p.) (Supplementary Table S1).

### 4.2. Gene Set Refinement

For the detailed transcriptomic analysis, a priori known gene list of neuro-regulatory and glycosylation pathways were used. For generating a unique list of gene markers associated with the neuroendocrine pathways a list was manually compiled from the Harmonizome open-source online database hosted by the Ma’ayan Laboratory of Computational Systems Biology (https://maayanlab.cloud/Harmonizome/ (accessed on 22 December 2022)) [80] and from the HGNC database (https://www.genenames.org/ (accessed on 22 December 2022), (EMBL-EBI, Cambridge, UK)). Rodent homologs for the human gene counterparts were derived from the vertebrate homologs data provided by the Mouse Genome Informatics (MGI) database (http://www.informatics.jax.org/homology.shtml (accessed on 22 December 2022) (The Jacksons Laboratory)) [81]. Similarly, a unique list of glycogenes was compiled by leveraging the GlycoGAIT database [82].

### 4.3. Functional Enrichment Analysis, Cellular Process Mapping and Network Visualization

Functional enrichment analysis of DEGs were conducted using g:Profiler [83] (https://biit.cs.ut.ee/gprofiler/ (accessed on 22 December 2022), (ELIXIR Infrastructre, UK)) and the ConsensusPathDB-human (CPDB—http://cpdb.molgen.mpg.de/ (accessed on 22 December 2022), (Max Planck Institute, Berlin, Germany)) [84]. The HGNC gene symbol of the DEGs were used as the query in the g:GOST functional profiling interface in g:Profiler using *Homo sapiens* as the organism species, g:SCS threshold as the significance threshold and the user-defined *p*-value threshold as 0.05 in the advanced options. Similarly, the “over-representation analysis” feature was used for the enrichment analysis using the HGNC gene symbol as the gene identifier in CPDB database. To facilitate the biological interpretation as well as to visualize the functionally grouped Gene Ontology, pathway term networks and associated markers, plugins ClueGo and CluePedia were utilised [85,86] within Cytoscape v.3.9 (http://www.cytoscape.org/ (accessed on 22 December 2022), (Institute of Systems Biology, Seattle, WA, USA)) [87].

## Figures and Tables

**Table 1:** Proposed molecular mechanism of action for psychedelic drugs. The target mechanism of action for each of the selected psychedelic drugs is manually integrated by curating published literatures. Mechanism of action is organized based on 4 major neurotransmitter signaling, which are well known in the literature to be affected by the psychedelic drugs. Images for each of the compounds are taken from the PubChem (light green colour indicates Hydrogen atom, blue colour for Nitrogen atom, red colour for Oxygen atom and the green colour for Chlorine atom). Up (↑) and down (↓) arrow indicates the increasing and decreasing levels of neurotransmitters in response to psychedelic molecules on each signaling pathways, respectively Abbreviations: 5-HT (serotonin), NMDA (N-methyl-D-aspartate).

Psychedelic Molecules	Structure	Serotonergic Signalling	Glutamate Signalling	Dopamine Signalling	Cholinergic/Adrenergic Signalling	References
DMT: N,N-dimethyltryptamine	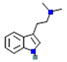	5-HT_2A_, 5-HT_1A_ and 5-HT_2c_ receptors (stimulatory)	↑ postsynaptic glutamate	↑ the release of dopamine as well as degradation	↓ Acetylcholine in corpus striatum	[10]
LSD: (5R,8R)-(+)-lysergic acid-N,N-diethylamide	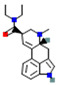	5-HT2 (stimulatory) and 5-HT1 (inhibitory). Also binds other 5-HT1 subtypes	↑ postsynaptic glutamate	agonist activity at dopamine D2 and D4 receptor	↑ Acetylcholine	[11]
Psilocybin	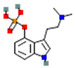	high affinity at 5-HT_2_ receptors (stimulatory)	↑ postsynaptic glutamate	↑ dopamine levels		[12]
Mescaline	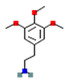	high affinity at 5-HT_2A._ receptors (stimulatory)	↑ postsynaptic glutamate	induced release of dopamine		[13,14]
5-HO-DMT: N,N-Dimethyl-5-Hydroxytryptamine	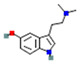	high affinity for the 5-HT_1A_ receptor (stimulatory)				[15]
5-MeO-DMT: 5-Methoxy-N,N-Dimethyltryptamine	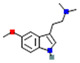	5-HT_2A_ >5-HT_2C_ >5-HT_1A_ receptors (stimulatory)				
Ketamine	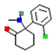	Inhibits serotonin uptake and thereby ↑ the concentration	Antagonizes NMDA and non-NMDA receptors	Inhibits dopamine uptake and thereby increases the concentration	Inhibition of catecholamines uptake, ketamine provokes a hyperadrenergic state (release of norepinephrine, dopamine, and serotonin).	[16]
Phencyclidine	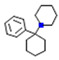	Inhibits serotonin uptake and thereby ↑ the concentration	Antagonizes NMDA	Inhibits dopamine uptake and thereby increases the concentration	Inhibition of norepinephrine reuptake and binds to muscarinic Ach receptor	[17]
MDMA: 3,4-Methylenedioxymethamphetamine	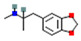	Enhanced serotonin release followed by substantial decrease		↑ dopamine release	↑ norepinephrine release	[18,19]

**Table 2:** Molecular function of neuro-regulatory and glycogene DEGs in response to ketamine and phencyclidine treatment. The table captures the selected DEGs that are 0.5 log fold differentially regulated (light, green-coloured boxes representing the down-regulated genes and the light, red-coloured boxes for the up-regulated genes). Only those data points that have significant representation of the neuro-regulatory and glycogene DEGs are selected for making the table with only including the genes that match the fold change filter criteria and are represented in at least 3 out of the 9 selected data points. For the full list of the DEGs refer Supplementary Tables S6 and S7.

Gene Symbol	GSE138802	GSE73799				GSE73799			
	Ketamine vs. Saline					Phencyclidine vs. Saline			
	Pre-Limbic Cortex	1 h	2 h	4 h	8 h	1 h	2 h	4 h	8 h
		Striatum				Striatum			
**Neuro-Regulatory Genes**									
**Kl Klotho** [Cleaved into: Klotho peptide]	**0.0**	**−0.5**	**1.1**	**0.0**	**0.0**	**1.2**	0.0	0.8	0.5
**Cdkn1a**—Cyclin-dependent kinase inhibitor 1	0.0	0.5	0.0	0.0	**−** 0.6	0.0	0.9	0.0	**−** 0.7
**Pomc**—Pro-opiomelanocortin (POMC) (Corticotropin-lipotropin)	0.0	**−** 0.5	0.0	0.0	0.7	**−** 0.6	0.0	0.0	0.6
**Klf4**—Krueppel-like factor 4	0.6	0.0	**−** 0.7	**−** 0.4	0.0	0.5	0.0	**−** 0.5	0.0
**Ncam1—**Neural cell adhesion molecule 1	0.0	0.0	0.0	0.0	**−** 0.9	0.0	0.9	0.0	**−** 1.1
**Aplp1—**Amyloid beta precursor like protein 1	0.0	0.0	0.0	0.0	**−** 0.9	0.0	0.8	0.0	**−** 1.1
**Scg5—**Neuroendocrine protein 7B2 (Secretogranin V)	0.0	0.0	0.0	0.0	**−** 0.8	0.0	0.8	0.0	**−** 1.0
**Ptprz1—**Receptor-type tyrosine-protein phosphatase zeta	0.0	0.0	0.0	0.0	**−** 0.8	0.0	0.7	0.0	**−** 0.9
**Cartpt—**Cocaine- and amphetamine-regulated transcript protein	1.9	0.0	0.0	1.0	0.0	0.0	0.0	0.8	0.0
**Gal—**Galanin peptides	5.4	0.0	0.0	0.9	0.0	0.0	0.0	0.8	0.0
**Oxt—**Oxytocin-neurophysin 1	7.7	0.0	0.0	1.6	0.0	0.0	0.0	1.5	0.0
**Avp—**Vasopressin-neurophysin 2-copeptin	7.6	0.0	0.0	1.1	0.0	0.0	0.0	1.3	0.0
**Chrna10—**Cholinergic receptor, nicotinic, alpha polypeptide 10	5.2	**−** 0.5	**−** 0.4	0.0	0.0	**−** 0.6	0.0	0.0	0.0
**Pde1b—**Calcium/calmodulin-dependent 3’,5’-cyclic nucleotide phosphodiesterase 1B	1.0	0.0	0.0	0.0	**−** 1.0	0.0	0.0	0.0	**−** 1.1
**Syn1—**Synapsin-1 (Synapsin I)	0.0	0.0	0.0	0.0	**−** 0.8	0.0	0.0	0.8	**−** 0.9
**Ppfia3—**Liprin-alpha-3	0.0	0.0	0.0	**−** 0.5	0.0	0.0	0.0	**−** 0.5	**−** 0.7
**Syt6—**Synaptotagmin-6 (Synaptotagmin VI)	2.0	0.0	0.0	**−** 0.6	0.0	**−** 0.4	0.0	**−** 0.6	0.0
**Col18a1—**Collagen alpha-1(XVIII) chain	0.6	0.0	0.0	0.7	0.0	0.0	0.0	0.7	0.0
**B3gat1—**Galactosylgalactosylxylosylprotein 3-beta-glucuronosyltransferase 1	0.0	**−**0.4	0.0	**−** 0.5	**−** 0.9	**−** 0.4	0.0	**−**0.4	**−** 1.2
**Fkbp5—**Peptidyl-prolyl cis-trans isomerase FKBP5	0.0	0.0	0.0	0.7	**−** 0.5	0.0	0.0	0.5	0.0
**Sytl2—**Synaptotagmin-like protein 2	**−** 0.7	**−** 0.5	0.0	0.0	0.0	0.0	0.0	0.0	0.5
**Gnas—**Guanine nucleotide-binding protein G(s) subunit alpha isoforms short	0.0	0.0	**−** 0.5	**−** 0.5	0.0	0.0	**−** 0.5	**−** 0.7	0.0
**Sstr5—**Somatostatin receptor type 5	0.0	0.0	**−** 0.4	**−** 0.5	0.0	0.0	**−** 0.5	**−** 0.7	0.0
**Glycogenes**									
**Hexb—**Beta-hexosaminidase subunit beta	*0.0*	*0.0*	*0.0*	*0.0*	**−** 0.8	0.0	0.8	**−** 0.8	**−** 0.8
**Kera—**Keratocan (KTN)	*0.0*	*0.0*	*0.0*	**−** 0.6	0.5	0.0	**−** 0.5	**−** 0.6	0.0
**Ogn—**Mimecan (Osteoglycin)	*0.0*	*0.0*	*0.0*	*0.0*	**−** 0.6	0.6	0.0	**−** 0.5	**−** 0.7
**Hs6st2—**Heparan-sulfate 6-O-sulfotransferase 2	0.5	*0.0*	*0.0*	*0.0*	**−** 0.8	0.0	0.0	**−** 0.8	**−** 0.9
**Alg6—**Dolichyl pyrophosphate Man9GlcNAc2 alpha-1,3-glucosyltransferase	*0.0*	−0.5	*0.0*	*0.0*	**−** 0.7	0.0	0.0	0.0	**−** 0.8
**Calr3—**Calreticulin-3 (Calreticulin-2)	*0.0*	−0.5	*0.0*	−0.6	0.3	0.0	0.0	−0.9	0.4
**Col9a2—**Collagen alpha-2(IX) chain	*0.0*	*0.0*	*0.0*	*0.0*	−0.5	0.0	0.0	−0.5	−0.6
**Mgat5—**Alpha-1,6-mannosylglycoprotein 6-beta-N-acetylglucosaminyltransferase A	*0.0*	−0.6	−0.5	*0.0*	−0.4	0.0	−0.8	0.4	0.0
**B3gat1—**Galactosylgalactosylxylosylprotein 3-beta-glucuronosyltransferase 1	*0.0*	−0.4	*0.0*	−0.5	−0.9	−0.4	0.0	−0.4	−1.2
**Alg3—**Dol-P-Man:Man(5)GlcNAc(2)-PP-Dol alpha-1,3-mannosyltransferase	*0.0*	*0.0*	*0.0*	0.6	*0.0*	0.0	0.0	0.5	−0.5
**Glt8d2—**Glycosyltransferase 8 domain-containing protein 2	−0.9	−0.6	*0.0*	*0.0*	*0.0*	−0.5	0.0	0.0	0.0
**Tpst2—**Protein-tyrosine sulfotransferase 2	*0.0*	*0.0*	*0.0*	*0.0*	−0.8	0.0	0.7	0.0	−0.9
**Dcn—**Decorin (Bone proteoglycan II)	*0.0*	*0.0*	1.0	*0.0*	−0.6	0.0	0.0	0.0	−0.6
**Bgn—**Biglycan (Bone/cartilage proteoglycan I)	0.8	*0.0*	*0.0*	*0.0*	−0.7	0.0	0.0	0.0	−0.7
**Ndst1—**Bifunctional heparan sulfate N-deacetylase/N-sulfotransferase 1	*0.0*	0.5	*0.0*	*0.0*	−0.6	0.0	0.0	0.0	−0.8
**Tmtc4—**Protein O-mannosyl-transferase TMTC4	*0.0*	*0.0*	−0.7	*0.0*	*0.0*	0.0	−0.9	−0.5	0.0
**Ugt1a2—**UDP-glucuronosyltransferase 1-2	*0.0*	−0.3	*0.0*	−0.8	*0.0*	0.6	0.0	−0.7	0.0

## Data Availability

Not applicable.

## Supplementary Materials

The following supporting information can be downloaded at: https://www.mdpi.com/article/10.3390/ijms24021200/s1.
