# Supplementary material for: Transcriptomic Analysis of Glycosylation and Neuroregulatory Pathways in Rodent Models in Response to Psychedelic Molecules
Source: Int J Mol Sci. 2023 Jan 7;24(2):1200. doi: 10.3390/ijms24021200 (PMC9867456; doi:10.3390/ijms24021200)
Supplement: Supplementary file 1 [file ijms-24-01200-s001.zip › Supplemental_Figures.pdf]

Supplementary Figures:

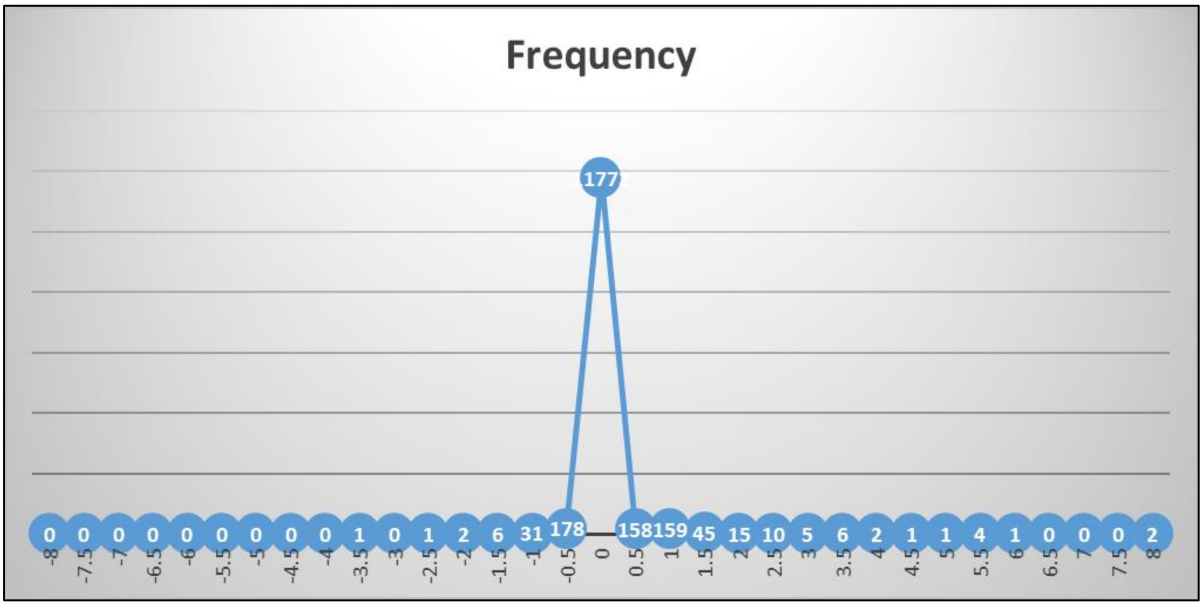

Supplementary Figure S1: Bar chart depicting the fold change distribution of DEGs belonging to the prior-knowledge gene set category which are differentially regulated in response to the psychedelic like molecules. The chart was created using the Histogram feature available under the Data Analysis tool in Microsoft Excel.
